# Using an Existing DICOM Infrastructure to Enhance the Availability, Quality, and Efficiency of Imaging Throughout the Healthcare Enterprise

**DOI:** 10.1007/s10278-018-0118-3

**Published:** 2018-09-04

**Authors:** Dan Kayhart

**Affiliations:** Marshfield Clinic Health System, 1000 North Oak Ave, Marshfield, WI 54449 USA

**Keywords:** Enterprise imaging, Point-of-care imaging, Encounter-based imaging, Nonradiology imaging, Image management, PACS, VNA, DICOM

## Abstract

Managing the capture, label, and storage of medical imaging performed by nonradiology departments at the point of care has become a core issue for many health systems. In contrast to the well-organized and controlled workflows enjoyed by radiology, nonradiology imaging and its associated workflows are often chaotic. Left on their own, many nonradiology departments simply fend for themselves, finding ways to capture and store their imaging that meets the critical need, while falling far short of the ideal. The focus of this study was to build and implement a software solution for nonradiology image management for a large, multi-specialty health system. Once developed, the solution was deployed and results were collected from users via survey and by comparison of available pre- and post-implementation statistics. The results show high rates of satisfaction among users, evidence of cost savings from decommissioning redundant systems, staff time savings, and fewer storage failures.

## Background

As imaging technology continues to advance and medical imaging devices, such as ultrasound machines, have become more affordable, many organizations are incorporating imaging into their everyday practice of medicine. This rise in the volume and variety of nonradiology imaging done in the context of patient encounters brings many challenges. Often nonradiology specialties find themselves and their image studies isolated from the same robust information technology (IT) support, electronic health record (EHR) integration, and image management tools available for radiology imaging. Furthermore, the unique needs and workflows of nonradiology practices often prevent the successful adoption of existing processes. As a result, these specialties typically devise their own workflows or purchase separate image management systems in an effort to meet their needs. This leads to image studies with incorrect or incomplete patient and procedure metadata, studies stored into disparate systems, limited accessibility, and little or no integration with the EHR. In some cases, care teams are using imaging equipment as a tool for patient care without the ability to store a study for later viewing.

Marshfield Clinic Health System (MCHS) is a multi-specialty group practice with over 700 providers throughout 50 different locations in rural central and northern Wisconsin. With 86 different specialties and subspecialties, MCHS has experienced the challenges of nonradiology imaging first-hand. Seeing the acute needs of care teams, MCHS sought a comprehensive strategy for ordering, capturing, storing, and viewing nonradiology medical imaging. This study examines the methods and results MCHS employed in an effort to drive improvements in nonradiology imaging quality, access, and cost.

## Methods

MCHS approved a project to develop a software tool to support standard methods and workflows for labeling and storing nonradiology images that could facilitate centralized access and image viewing within the EHR. A secondary goal of the project was to consolidate imaging resources in a way that other imaging systems in place for some specialties could be eliminated. This allowed MCHS to maximize the investments made in its Digital Imaging and Communications in Medicine (DICOM) infrastructure and reduce cost.

The primary product of this project was a new application and system operating under the name “Image Locker.” Image Locker is a software application developed to facilitate attachment of patient, encounter and procedure data to nonradiology images while taking advantage of existing DICOM tools. Image Locker allows clinicians to specify procedure information such as modality, study description, anatomy, and side for the image study, either before the images have been captured or after they have been sent to the Vendor Neutral Archive (VNA). The primary efficiency of the application is that it delivers patient and encounter details automatically to the user from the pre-existing appointment or admission for use with an imaging order. This is accomplished through an application-programming interface (API) and Health Level 7 (HL7) interface connections to the EHR.

**Fig. 1:**
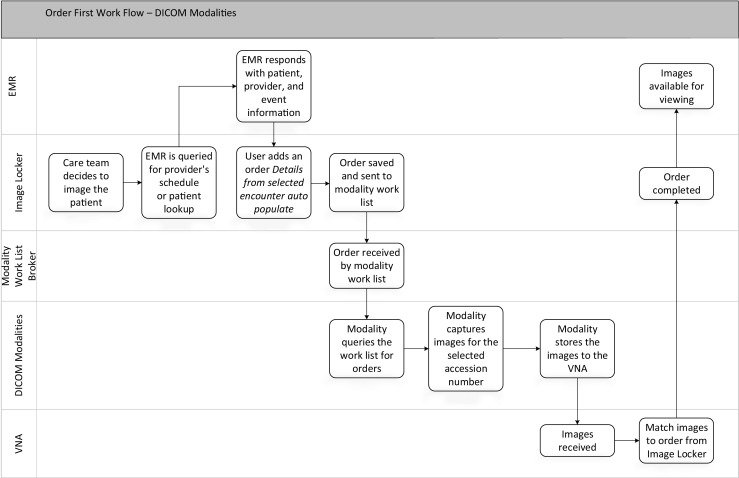
Order first work flow—DICOM modalities

Within Image Locker users can select a provider and location from a configurable list built for their department-specific care team. Once a provider is selected, the application queries the EHR for schedule data for that provider and displays it within the application. On each line of the schedule is a button that when clicked facilitates quick creation of an imaging order for the selected patient encounter. In situations where no appointment exists, another tab is available that allows the user to search for a patient by name or identification number within the application and then specify encounter details along with the order details. When procedure details are specified in the method described above prior to the procedure, referred to as “order first” workflow (Fig. 1), the application sends an HL7 order message to a DICOM Modality Worklist (DMWL) where it can be queried and used by a DICOM modality. Once selected, the order is applied to the captured images and then the study is sent to the VNA where it is archived and made available for viewing within the EHR.

**Fig. 2:**
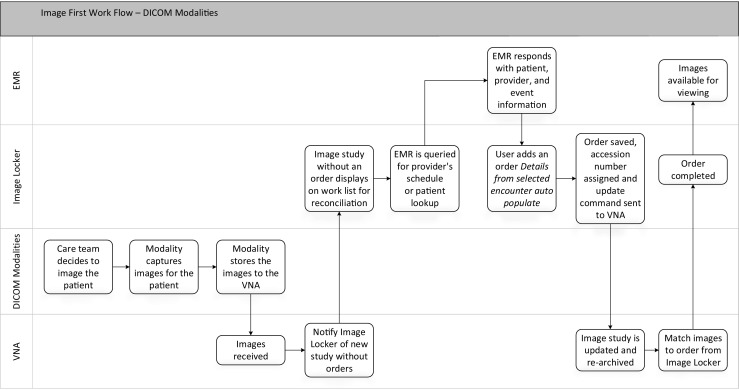
Image first work flow—DICOM modalities

In emergency situations or those in which the images may or may not need to be archived, a clinician can choose to capture the images on the modality first, referred to as “image first” workflow (Fig. 2). In this workflow, the unsolicited images are first captured at the modality and sent to the VNA, where an application plug-in checks if the sending device is flagged to require an Image Locker order. If Image Locker orders are required, the study is quarantined into a reconciliation database at the VNA and the plug-in notifies Image Locker that new images without an order have arrived from a particular modality. The notification alerts users that images are not yet archived or viewable in the EHR. Rules within Image Locker create a post-capture reconciliation worklist that displays unverified studies for the care team in the home view of the application. A care team in this context is a group of clinical users working together in a particular department and facility. To reconcile the study, a care team member can associate an encounter from their schedule right within the application and add the appropriate procedure information just as they would if using the order first method prior to image capture. Once the metadata is added, the system integrates with the VNA to automatically run a series of built in commands that coerce the DICOM tags of the study and fill in the correct values to add context. Finally, the study is processed at the archive a second time, (basically re-sending the study to itself as if it were received a second time from the modality). With the metadata complete and an Image Locker order to match to, the updated study completes verification and stores in the VNA, making the images viewable in the EHR once the correct metadata is applied. An additional set of rules and escalation logic ensures each care team addresses the unfinished image studies on their list within a set timeframe. If an image study remains unreconciled, clinical practice administration is automatically notified of the orphaned study.

**Fig. 3:**
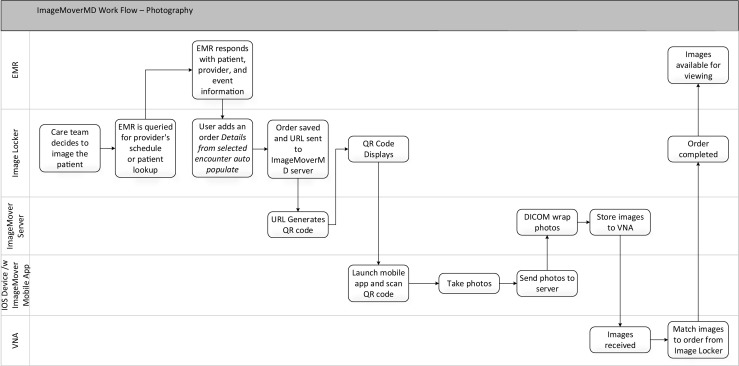
ImageMoverMD work flow—photography

For medical photography studies, the application integrates with the “ImageMoverMD” vendor solution (www.imagemovermd.com) via URL (Fig. 3). This URL generates and displays a QR code that is scanned on the clinician’s mobile device to tie the patient metadata to the pictures captured on the device. After capture, pictures are sent to the ImageMoverMD server that DICOM wraps and forwards the study to the VNA for archiving and viewing. At MCHS, the URL is generated by Image Locker using the order first workflow to capture the required metadata. The QR code supplied by ImageMoverMD then appears within the Image Locker application in an i-frame for scanning. An image first workflow is not currently supported for photography because the QR code is required for secure capture and upload from the mobile device.

MCHS has also purchased a few licenses and vendor software packages to DICOM-wrap the images or PDF’s for other non-DICOM modalities such as visual fields and endoscopy. A possible future enhancement to Image Locker is being investigated to add native DICOM wrapping within the application that will allow a user to upload additional types of non-DICOM medical images to the VNA from their workstation. The MCHS ad hoc imaging strategy committee has also put in place a review process for new imaging devices to ensure that a DICOM capable device is chosen whenever possible. This strict adherence to proven standards such as DICOM and HL7 is intended to foster an environment where all images can be stored, viewed, sorted, and verified in the same manner from a single location in the EHR. It also ensures interoperability with other systems, offering the health system flexibility when choosing other software solutions. This approach does carry some cost but in the experience of MCHS, it has proven less expensive than purchasing and maintaining disparate systems to manage different types of proprietary imaging.

As of December 2017, Image Locker has been implemented in numerous specialties at MCHS where a variety of modalities are used. Examples of specialties include: Ophthalmology, Emergency Medicine, Intensive Care, Cardiology, Rheumatology, Orthopedics, Physical Therapy, Ambulatory Surgery, Otolaryngology, Urology, Women’s Health, Dermatology, Telemedicine, General Surgery, Plastic Surgery, Pain Management, Physical Medicine, Internal Medicine, Endocrinology, Infectious Disease, Neurology, Oral Surgery, Podiatry, Nursing Home Services, Orthotics/Prosthetics, Pediatrics, Pulmonology, Wound Care, Sports Medicine, Speech Language Pathology, and Primary Care. Future implementation is planned for Gastroenterology.

## Results

As a result of the new system’s utilization of an existing radiology infrastructure, MCHS was able to decommission a separate system for Ophthalmology. There are also plans to decommission another image management system currently in use for Gastroenterology procedures. The decommissioning of these systems saves the Health System money from the reduction of software licensing, upgrades, servers, and maintenance fees to vendors.

After the implementation of Image Locker, a survey was administered to just over 700 health care professionals utilizing the application. Of those, 98 survey recipients responded and offered valuable information about how the application is used and how it has been perceived on the floors. When asked to rate their overall satisfaction with the application, 77% of respondents answered they are satisfied or highly satisfied. Half of respondents indicated they use the application at least weekly and 58% indicated that the new system saves them time, with “up to 5 minutes per day” being the most common response. A small timing study was also conducted with six volunteers from various MCHS clinics in rheumatology, dermatology, and oral surgery. A stopwatch was used to measure the time it took volunteers to label and upload images using the old processes that were in place in the past compared with Image Locker and ImageMoverMD. Each of the participants was an experienced technician in their specialty. The results showed an average time savings of 2.5 min per image study. For photo studies alone, this equates to over 800 h of time saved annually, assuming 20,000 studies per year. Survey respondents stated the value of the application to their practice in the following statements. “It helps document clinical scenarios that are difficult to put into words.” “It is much more efficient and provides greater resolution of pictures which assists in patient skin monitoring.” “The images are directly linked to patient and this prevents user errors (entering in names/numbers/birthdays incorrectly).” “Patient information entered into imaging machines accurately.”

In addition to these results, far fewer nonradiology imaging studies require support and intervention than in the past. Image Locker enforces high standards for metadata integrity and ensures that images store through a post-capture reconciliation worklist and associated escalation logic that notifies a manager or administrator when studies are not able to be stored in a timely manner. Objective evidence illustrates this when comparing pre- and post-implementation statistics from one orthopedics department. In a 6-month period extending from April 1 to September 30, 2016, there were 60 studies that failed to store due to demographic or accession number verification errors. In the same period of 2017, there were 0 failures for the same department. Similar results are evident for other nonradiology departments capturing and storing images in various ways before the implementation.

## Discussion

Thus far, results of this case study have shown positive financial, technical, and clinical outcomes that have the potential for improving both care quality and efficiency. High marks of satisfaction and positive comments from users indicate an appreciation of the improved ability to capture and store nonradiology images at the point of care. This speaks to a fulfillment of some of the unmet needs clinicians, particularly specialists, often have to convey or record information more clearly through an image [1, 5]. Consolidation of image capture workflows and use of the existing DICOM infrastructure has provided an environment where all medical imaging can be accessible from one area of the patient’s electronic health record. That accessibility affords providers more opportunities to collaborate efficiently using images [2]. Improvements to indexing and the ability to compare images and track changes over time have profound positive impact as well, particularly with imaging that is commonly non-DICOM such as photography [6]. This consolidation adds the benefit of cost savings by reducing overhead from supporting redundant technologies and overly cumbersome workflows.

While the Image Locker and ImageMoverMD software tools have provided a strong framework for enforcing high standards and virtually eliminating situations where studies cannot be stored or found due to missing metadata, there remains a need for human intervention and other software tools to correct misfiling errors. The most common errors are those caused by choosing an incorrect accession number from the work list, or mistakenly capturing two patients’ images on a single accession number. These scenarios necessitate wrenching of the study DICOM tags or in some cases splitting a single study into two. Third party image qc software tools, or built-in capabilities within the VNA or PACS are often utilized to make those corrections. Typically, the errors are reported by users to the helpline and triaged to an appropriate PACS or IT resource with the skills and knowledge to correct the problem.

A strong imaging governance structure that promotes strict adherence to proven technologies and standards for integration with available imaging modalities, archives, and EHRs is a key factor that has driven the implementation of an enterprise-wide imaging strategy at MCHS. An ad hoc imaging strategy committee consisting of key stakeholders from various areas of the health system was formed to develop the overall trajectory and make decisions related to imaging strategy. Members include representatives from IT, Health Information Management (HIM), various providers with high interest in imaging, and the director of the Institute for Quality, Innovation, and Patient Safety (IQIPS). The committee convenes on an as needed basis to identify and discuss strategic initiatives and works to promote the advancement of projects, tools, policies, and procedures that enhance patient care through imaging. Roth, Lannum, and Joseph point out that “Like electronic health record (EHR) governance, both the enterprise imaging program governance bodies and the stakeholders should understand that the intent of imaging governance is towards long-term strategic enablement, not erecting roadblocks.” [3] In the spirit of that same intent, the adoption of proven standards has enabled a long-term strategic trajectory for MCHS, which is supported and enforced by the Image Locker software.

A key to successful adoption of Image Locker by clinical teams is the flexibility of its workflows. This flexibility allows patient and study metadata to be applied either before or after images are captured and archived; with or without a pre-defined appointment or encounter from the EHR. Cram, Roth, and Towbin point out that different specialties are likely to favor different workflow approaches, but as long as those workflows can be controlled in such a way as to achieve a set of common goals and outcomes, the clinical needs can still be met [4]. These goals include matching images with an appropriate patient and provider interaction, modality, study description, anatomy, facility, and correct demographics. With that set of common goals in mind, Image Locker manages the convergence of images, interactions, and metadata to meet the needs of many different specialists who interact with patients across a wide spectrum of environments and circumstances.

## Conclusion

The challenges imposed by an ever-changing medical imaging landscape and the increasing need for better management and integration of images from nonradiology departments are complex. However, there is great opportunity to develop solutions that can simplify this complexity and improve the ways in which clinicians capture, label, store, view, and share medical images. By taking advantage of proven standards and existing tools, a flexible workflow solution can meet the needs of clinicians, increase efficiency, and improve the quality of care patients receive while reducing cost. A successful enterprise imaging strategy, combined with implementation of a robust platform of software tools for managing workflows and outcomes can make a substantial positive impact, bringing order and simplicity to an otherwise chaotic situation.
